# Treatment of Complex Cutaneous Leishmaniasis with Liposomal Amphotericin B

**DOI:** 10.3390/pathogens10101253

**Published:** 2021-09-28

**Authors:** Maria Ubals, Pau Bosch-Nicolau, Adrián Sánchez-Montalvá, Fernando Salvador, Gloria Aparicio-Español, Elena Sulleiro, Aroa Silgado, Antoni Soriano-Arandes, Maria Espiau, Berta Ferrer, Diana Pou, Begoña Treviño, Israel Molina, Vicente García-Patos

**Affiliations:** 1 Department of Dermatology, Hospital Universitari Vall d’Hebron, Universitat Autònoma de Barcelona, 08035 Barcelona, Spain; mariaubalscazorla@gmail.com (M.U.); glaparic74@gmail.com (G.A.-E.); vgarciapatos@gmail.com (V.G.-P.); 2 Doctoral Programme in Medicine and Translational Research: International Health Track, Facultat de Medicina, Universitat de Barcelona, 08036 Barcelona, Spain; 3 Department of Infectious Diseases, Hospital Universitari Vall d’Hebron, PROSICS Barcelona, Universitat Autònoma de Barcelona, 08035 Barcelona, Spain; pau.boschnicolau@gmail.com (P.B.-N.); medinano@yahoo.es (F.S.); israelmolina@ymail.com (I.M.); 4 Facultat de Medicina, Universitat Autònoma de Barcelona, 08035 Barcelona, Spain; 5 Department of Clinical Microbiology, Hospital Universitari Vall d’Hebron, PROSICS Barcelona, Universitat Autònoma de Barcelona, 08035 Barcelona, Spain; esulleir@vhebron.net (E.S.); aroa.silgado@vhir.org (A.S.); 6 Department of Paediatrics, Hospital Universitari Vall d’Hebron, Universitat Autònoma de Barcelona, 08035 Barcelona, Spain; asoriano@vhebron.net (A.S.-A.); mespiau@vhebron.net (M.E.); 7 Department of Pathology, Hospital Universitari Vall d’Hebron, Universitat Autònoma de Barcelona, 08035 Barcelona, Spain; bferrer@vhebron.net; 8 Tropical Medicine and International Health Unit, Drassanes-Vall d’Hebron, PROSICS Barcelona, 08035 Barcelona, Spain; d.pou@vhebron.net (D.P.); btrevino.bcn.ics@gencat.cat (B.T.)

**Keywords:** complex cutaneous leishmaniasis, cutaneous leishmaniasis, liposomal amphotericin B, leishmania, systemic therapy

## Abstract

Background: There is no consensus for the best treatment of complex cutaneous leishmaniasis (CL). We aimed to describe a cohort of CL, focusing on liposomal amphotericin B (L-AmB) treatment outcome. Methods: We performed a retrospective study in Vall d’Hebron University Hospital (Barcelona, Spain). All patients with parasitologically proven CL diagnosed from 2012 to 2018 were included. Results: The analysis included 41 patients with CL. The median age was 39 years (IQR 12- 66); 12 (29%) were children, and 29 (71%) were men. Regarding treatment, 24 (59%) received local treatment, whereas 17 (41%) had complex CL and were offered intravenous systemic treatment. Sixteen patients received L-AmB; eight (50%) had adverse events, and three (19%) discontinued treatment for safety reasons. All cases were considered cured within the first year post-treatment. Conclusions: L-AmB for complex CL showed no treatment failures, offering an alternative treatment option for patients with complex CL. Clinicians should pay close attention to the potential adverse events of L-AmB and adopt an active drug safety surveillance scheme to rapidly detect reversible side effects.

## 1. Introduction

Cutaneous leishmaniasis (CL) is a sand-fly-borne disease caused by 1 of 23 different pathogenic species of the genus *Leishmania.* The World Health Organization (WHO) estimates that one million cases of CL occur every year worldwide [1,2]. The protozoan parasites in promastigote form are transmitted to susceptible mammalian hosts by female sand flies (*Phlebotomus, Lutzomyia)*. According to the geographic distribution, CL can be classified into New World CL (American Continent, transmitted by *Lutzomyia* sand flies) and Old World CL (Mediterranean basin, northeast Africa, Middle East, South Asia, transmitted by *Phlebotomus* sand flies). The most common species that cause New World CL are *L. V (Viannia). brazilensis, L. V. panamensis* and *L.V. guayanensis*; and Old World CL is generally caused by *L. L (Leishmania). infantum*, *L. L. major* and *L. L. tropica*. In the Mediterranean basin, the most common causative specie of CL is *L. Infantum,* and its usual reservoir is the dog. However, in recent years, an increasing incidence of other species has been observed due to returning travelers and migration movements (*L. tropica, L. donovani and L. major*) [3].

After a careful diagnostic evaluation, management should be individualized based on the classification in simple or complex CL. Complex CL is defined by a high risk of mucosal involvement, numerous or very large lesions, disseminated CL, lesions located in places with difficult access to local treatment, immunosuppression or clinical failure of local therapy [4]. Patients with simple CL can be offered local therapy or observation without treatment. However, the Infectious Diseases Society of America (IDSA) guidelines recommend systemic treatment for patients with complex CL [4]. 

Systemic pentavalent antimonials (Sb^v^) have shown cure rates of 85–90% in studies on Old World CL and are currently considered the ‘gold standard’ comparator drug for evaluating the efficacy of new therapies [5]. However, this treatment is frequently associated with severe adverse events (i.e., cardiotoxicity, elevation of liver and pancreatic enzymes, and bone marrow suppression), and up to 25% of patients may discontinue treatment due to safety reasons [6]. Alternative systemic treatment options for complex CL are intravenous liposomal amphotericin B (L-AmB), intravenous or intramuscular pentamidine, oral miltefosine and oral azoles. Of them, only miltefosine has been granted FDA approval for this indication, based on recent clinical trials [7,8,9], although it is not commercially available in most European countries. Data regarding the efficacy and safety of L-AmB for Old World CL treatment are limited to scarce retrospective studies, although they showed promising efficacy results with cure rate up to 84% [10,11]. 

The scarcity of clinical information regarding systemic agents for the treatment of complex CL and heterogeneity of healing rates between studies challenges the assessment of the trade-off between the efficacy and toxicity of these treatments. In the case of L-AmB, for which no randomized controlled trials are available in the CL setting, data from the real-world use contribute to characterizing the patient profile that can benefit more from this treatment. In this study, we aim to characterize patients with CL treated in a tertiary hospital in Spain and assess the treatment outcomes in those receiving L-AmB according to the IDSA criteria for systemic treatment.

## 2. Results

### 2.1. Demographic and Epidemiologic Characteristics

Between 2012 to 2018, 41 patients were diagnosed with CL at Hospital Universitari Vall d’Hebron. The median age was 39 years (IQR 12-66), 12 (29%) were children younger than 18 years, and 29 (71%) were male. Sixteen patients (39%) were migrants (13 from Morocco, 1 from Bolivia, 1 from Oman, and 1 from Honduras); fourteen (34%) acquired the infection during an international trip (10 in Morocco, 2 in Thailand, 1 in Turkey, and 1 in Bolivia). Nine (22%) patients, all autochthonous, had an immunosuppressing condition (6 anti-TNFα therapy recipients, 1 HIV infection, and 2 primary CD4 deficiency). Eighteen cases had no history of travel or immunosuppression. More data are shown in Table 1.

### 2.2. Diagnostic Methods and Classification

The median diagnostic delay was five months (IQR 2-8.25). Of the 32 cases analyzed for histopathology, 27 (84%) had findings that confirmed CL diagnosis. Of the 38 samples tested using microbiology techniques, 34 (89%) had a positive result (Table 1). The etiological agent was studied in 34 of 41 confirmed cases (83%): 21 (62%) patients had *L. infantum* infection, and 9 (26%) patients had *L. major* infection. The remaining 4 (12%) had insufficient DNA for testing.

**Figure 1:**
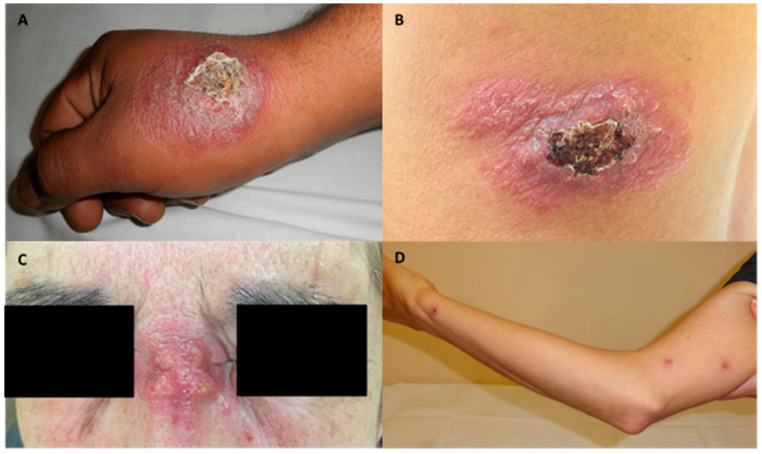
Images of four ulcers from different patients with complex features. (**A**): Hyperkeratotic and ulcerated plaque on the right hand from a patient with multifocal cutaneous leishmaniasis. (**B**): Hyperkeratotic 15 centimeters- plaque on the back. (**C**): Indurated plaque at the nasal root. (**D**): Subcutaneous nodules due to local dissemination on the right arm.

Twenty-four (59%) patients presented simple CL, while 17 (41%) patients had complex CL: 9 were immunocompromised, 2 had mucosal involvement, 3 multifocal disease, and 3 were large size ulcers. Lesions from some patients of the cohort are shown in Figure 1. Of the 16 patients with complex CL who received treatment with L-AmB, 7 had *L. infantum* infection, 5 *L. major* infection, and species identification could not be conducted in 4 patients.

### 2.3. Treatment Outcomes

Patients with simple CL received local treatment (more information in Supplementary Table S1). Systemic therapy with L-AmB was initiated in 16 of 17 patients with complex CL: 13 completed the 5-day regimen, reaching a cumulative dose of 20 mg/kg, and 3 had to discontinue treatment due to adverse events with at least a cumulative dose of 16 mg/kg. All 16 were cured 3 months after treatment completion. All patients showed good progression, and no relapses were reported at 12 month follow-up. 

One exceptional case with disseminated CL in the context of visceral leishmaniasis (VL) was treated with pentamidine. It was administered in dosages of 4 mg/kg/day on alternate days for a total of 15 doses, not exceeding 300 mg daily.

### 2.4. Treatment Safety

**Figure 2:**
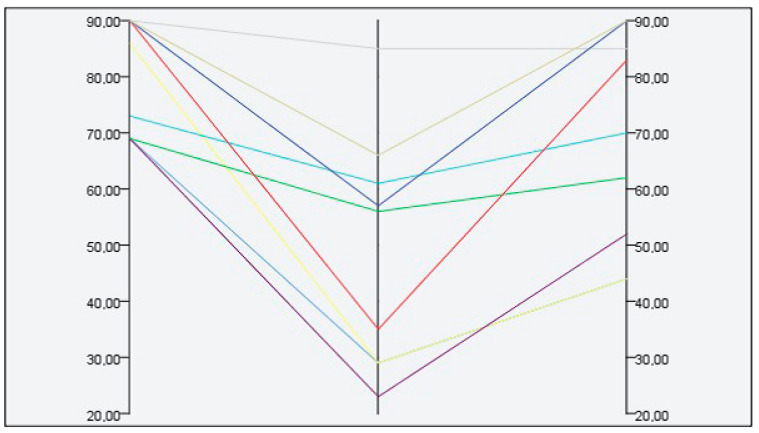
Kidney function test at different time points in patients treated with L-AmB. Glomerular filtrate (mL/min/1.73m^2^), time points—before treatment, immediately post-treatment and 1 month after treatment onset.

No serious adverse events were detected in the group of patients treated locally. However, 8 (50%) of patients who received L-AmB had an adverse reaction (Table 2), all grade III or more, including 4 patients with acute kidney failure, 2 of them requiring hospital admission (detailed information of cases is depicted in Supplementary Table S2). All adverse events resolved after treatment discontinuation. Figure 2 shows the glomerular filtrate measured at three time points (before, immediately post-treatment, and 1 month after the therapy) in patients treated with L-AmB. The median decrease in the glomerular filtrate was 15.5 mL/min/1.73 m^2^ (IQR 10.5–27.75). All patients recovered normal kidney function three months after treatment.

## 3. Discussion

In this cohort of 16 patients with complex CL, 5 day treatment with L-AmB was associated with good clinical outcomes, we observed complete clinical healing at 3 months, and lack of relapse at 12 months of follow-up. Species identification was in concordance with geographic exposure, with autochthonous *L. infantum* being the main species in our cohort (62%). The high frequency of this species, more susceptible to L-AmB than others [10], may have contributed to the high healing rates observed in our cohort. Despite the high effectiveness of L-AmB in patients with complex CL, treatment resulted in high frequency of adverse events, either infusion-related events or nephrotoxicity. 

Most CL self-heal over a period that may range from 2 to 18 months [12]; therefore, a conservative approach with local therapy should be appropriate in most CL infections, particularly those that are simple CL forms in immunocompetent hosts. However, patients with complex CL, such as the 16 cases reported in this analysis, may require systemic therapy to accelerate the healing of lesions, improve scarring and avoid the spread of the disease. 

Like in many other neglected diseases, the poor quality of clinical evidence and lack of strong consensus on CL treatment [13,14] challenges making therapeutic decisions in the daily management of these patients. The main reasons for prescribing systemic treatment in our cohort included reducing scarring in large ulcers located in places with difficult access to local treatment, and immunosuppression. While this approach seems reasonable because it increases the delivery of the therapeutic agent to distant sites of potential spread, it is unclear to what extent all immunosuppressed patients would require systemic therapy. A recent review of 49 patients receiving therapy with TNF-α blockers (57.1% and 10.2% with cutaneous and mucocutaneous leishmaniosis, respectively) showed positive outcomes after systemic therapy—mainly with L-AmB—and discontinuation of the TNF-α blocker therapy until clinical resolution [15]. However, the immune response against *L. infantum* is complex, and not all immunosuppressed patients might be at similar risk of complex CL. A careful evaluation of the accrued immunosuppression over time, mechanism of action of the immunosuppressant medication and consequence of removing the immunomodulation drugs helps to guide the management of these patients. 

Sb^v^ have been widely used for the treatment of complex CL. They have a high cure rate, but the treatment is long, with frequent adverse events (25% patients require treatment discontinuation), and regular clinical, cardiac and laboratory monitoring is mandatory [16]. L-AmB and Sb^v^ show comparable efficacy for treatment of complex CL [17]. However, L-AmB treatment is a 5 day course therapy, compared with the 3 week course treatment with Sb^v^ drugs. A drawback with using L-AmB is its high cost, but the reduction in the number of follow-up visits, hospitalization days and the avoidance of blood tests and electrocardiograms required to monitory Sb^v^ toxicity make L-AmB cost beneficial in industrialized countries [18]. Miltefosine is an oral and well-tolerated therapy; however, clinical cure responses range from 50% to 90% depending on the species involved, and it is a still foreign unavailable medication in most European countries [7,19]. Lastly, Pentamidine is a second-line treatment, recommended when there is a therapeutic failure or in special situations, given its safety profile [5].

The main challenge of making therapeutic decisions regarding treatment with L-AmB is the trade-off between the risk of CL progression and the toxicity associated with L-AmB. In our cohort, half of the patients experienced moderate toxicity events. The most common adverse events were acute infusion-related events, which are associated with rapid infusion of L-AmB [20] and may occur in a frequency of up to 20% [11,21], and nephrotoxicity, which has been observed in up to 45% of cases [11]. Adverse events are reversible after discontinuation of L-AmB and medical treatment with intravenous hydration. In our cohort, L-AmB was administered with intravenous hydration and was slowly infused in order to mitigate nephrotoxicity and acute-infusion reactions.

Our study is limited by the retrospective design and the small sample size, but it provides real-world information on complex CL treated with L-AmB in the Mediterranean basin, currently lacking in the literature. Taken together, our results confirm the effectiveness of L-AmB for treating complex CL. However, the frequency of adverse events such as nephrotoxicity in healthy patients as a consequence of treating a non-lethal condition raises important concerns. Our results highlight the need for carefully weighing the risks and benefits of this systemic therapy and considering the broad repertoire of local therapies [4] before initiating a systemic agent. Furthermore, there is an urgent need to provide clinicians with evidence-based information regarding the type of patient who is more suitable for systemic treatment with L-AmB and progress marketing of alternative systemic agents. 

## 4. Materials and Methods

### 4.1. Patients’ Selection and Data Collection

We retrospectively analyzed all cases of microbiologically proven CL from January 2012 to December 2018 diagnosed at Hospital Universitari Vall d’Hebron (Barcelona, Spain).

The diagnosis of CL was based on clinical suspicion, with laboratory confirmation by either histopathology findings compatible with amastigotes and/or the presence of molecular test positive for Leishmania spp. analyzed with real-time polymerase chain reaction (PCR). A duplex real-time PCR technique was performed following the protocol described by Mary et al. [22] with some modifications. The protocol includes two targets, a specific region of leishmania spp. Kinetoplast DNA and human RNase P gene as internal control. A sample was considered positive for *Leishmania* DNA when the threshold cycle (Ct) for the *Leishmania* target was <40 and negative when did not detect the specific target but detected internal amplification.

We reviewed electronic health records to gather demographic and clinical information of the patients, including age, gender, country of origin, history of immunosuppressive conditions, concomitant medication, travel history, lesion size and location, diagnostic methods, treatment received, adverse events and outcome. 

### 4.2. Treatment Criteria

According to the hospital procedures, systemic treatment with L-AmB (Ambisome®, Gilead Sciences, Inc, Madrid, Spain) was offered based on the presence of multiple lesions (≥5), large individual skin lesion (≥5 cm), local dissemination (satellite lesions, large lymphadenopathy or subcutaneous nodules), CL caused by *Leishmania* species associated with increased risk for mucosal leishmaniasis, and lesion on the face, joints or genitalia (places with difficult access to local treatment). Additionally, immunocompromised hosts or patients with treatment failure to local therapy were offered systemic treatment [4]. L-AmB is an approved course treatment in Spain for VL and fungal infections; the physicians prescribed L-AmB off-label as part of a compassionate use for complex CL treatment. L-AmB was given daily for five consecutive days at a dose of 4 mg/kg/day in a 500 mL glucose 5% and infused over 3–4 h. The total cumulative dose for each patient was 20 mg/kg. Active safety monitoring assessment protocols in place included blood cell count and basic biochemistry tests before and after treatment completion. 

### 4.3. Outcome Criteria

Based on hospital protocols, patients with CL were followed for 12 months after treatment (twice a month until re-epithelialization and every three months thereafter). The clinical outcome was assessed at three months after treatment and classified as (1) healing (the ulcer was completely re-epithelialized), (2) partial response (≥50% decrease in the largest diameter of the main ulcer), and (3) treatment failure (<50% decrease in the largest diameter of the main ulcer). Relapse was defined as a deterioration of skin lesion after a period of improvement within the 12 months after treatment completion. The relapse-free cure at 12 months was chosen as complementary endpoint.

### 4.4. Statistical Analysis

We performed a descriptive analysis regarding epidemiological data, clinical manifestations and treatment outcomes. Qualitative variables were defined as absolute number and percentages and quantitative as the median and interquartile range (IQR), defined as the 25th and 75th percentiles. No hypothesis tests were performed.

### 4.5. Ethics Statement or Compliance with Ethical Standards 

The study protocol was approved by the Ethical Review Boards of Hospital Universitari Vall d’Hebron (Barcelona, Spain), approval number: EOM(AG)016/2021(5809). An exemption from obtaining informed consent was granted. Procedures were performed in accordance with the ethical standards laid down in the Declaration of Helsinki as revised in 2013.

## 5. Conclusions

Our experience shows high cure rates for complex CL with systemic L-AmB. Although adverse events are common, they are reversible and can be anticipated with an active safety monitoring assessment strategy. Considering the scarcity of therapeutic options for complex CL, L-AmB is currently convenient in a selected group of patients with complex CL when the benefit outweighs the risks because the course is short and tolerable. However, further research with alternative drugs is urgently needed.

## Figures and Tables

**Table 1:** Baseline characteristics of study participants.

**COMPLETE CL COHORT DATA**	**N = 41**
**Demographic data**	
Age (years), median	39 (IQR 12–66)
Gender (male)	29 (70%)
History of travel	14 (34%)
Autochthonous infection	25 (61%)
Immunosuppressing condition	9 (22%)
**Diagnostic method**	
Histopathological findings (n = 32)	27 (84%)
Positive PCR (n = 38)	34 (89%)
**Species identification (n = 34)**	
*L. infantum*	21 (62%)
*L. major*	9 (26%)
Indeterminate result	4 (12%)
**Type of CL**	
Simple CL	24 (59%)
Complex CL	17 (41%)
Immunocompromised	9
Mucosal involvement	2
Multifocal	3
Large size	3
**L-AMB TREATMENT COHORT DATA**	**N = 16**
**Demographic data**	
Age (years), median	44 (IQR 35–66)
Gender (male)	15 (94%)
History of travel	6 (38%)
Autochthonous infection	9 (56%)
Immunosuppressing condition	8 (50%)
**Diagnostic method**	
Histopathological findings (n = 14)	11 (79%)
Positive PCR (n = 14)	12 (86%)
**Species identification**	
*L. infantum*	7 (44%)
*L. major*	5 (31%)
Unknown species	4 (25%)

CL: cutaneous leishmaniosis, IQR: interquartile range (25th and 75th percentile).

**Table 2:** Adverse events related to L-AmB treatment.

	**N = 16**
**Type of adverse events**	8 (50%)
Infusional reaction	3 (19%)
Acute kidney failure, glomerular filtrate (mL/min) < 50%	4 (25%)
Nausea/vomiting	4 (25%)
Diarrhea	1 (6%)
Fever	1 (6%)
**Severity of adverse events**	
Grade I–II	0
Grade III	6
Grade IV	2

## Data Availability

The data presented in this study are available on request. The data are not publicly available due to privacy.

## Supplementary Materials

The following are available online at https://www.mdpi.com/article/10.3390/pathogens10101253/s1, Table S1: Local treatment and evolution of simple CL cases, Table S2: Age, comorbidities, concomitant medication, type and grade of adverse events presented in the cohort of complex CL treated with L-AmB.
