# Supplementary material for: Treatment of Complex Cutaneous Leishmaniasis with Liposomal Amphotericin B
Source: Pathogens. 2021 Sep 28;10(10):1253. doi: 10.3390/pathogens10101253 (PMC8537943; doi:10.3390/pathogens10101253)
Supplement: Supplementary file 1 [file pathogens-10-01253-s001.zip › pathogens-1350467-supplementary.pdf]

pathogens

MDPI

Table S1. Local treatment and evolution of simple CL cases.

Local Therapy 24 (59%)	N=24	Effectiveness 3 months	Rescue Treatment	Effectiveness 12 months
Cryotherapy	8 (33%)	Cured		Cured
Intralesional Sb^{v}	5 (21%)	Cured		Cured
Combination previous	8 (33%)	3 (13%) treatment failure	1 Imiquimod 5%	Cured
			2 Fluconazol	Cured
Surgery	1 (4%)	Cured		Cured
Observation	2 (8%)	Cured		Cured
		1 (4%) Lost follow-up		1 (4%) Lost follow-up

Table S2. Age, comorbidities, concomitant medication, type and grade of adverse events presented in the cohort of complex CL treated with L-AmB.

Patient	Age	Comorbidity	Concomitant Medication	Adverse event	Grade	Finish Treatment	Hospitalization
1	49	Arterial hypertension Psoriatic arthritis	Antihypertensive Infliximab	Fever Nausea Diarrhea	III	Yes	No
2	66	Inflammatory bowel disease	Infliximab	Infusional reaction Acute kidney failure	IV	Yes	**Yes**
3	84	Arterial hypertension	Antihipertensive	Acute kidney failure	III	Yes	No
4	39	Inflammatory bowel disease	Infliximab Azatioprine	Nausea/Vomiting Acute kidney failure	IV	**No**	**Yes**
5	55	-	-	Infusional reaction	III	Yes	No
6	66	Inflammatory bowel disease	Infliximab	Infusional reaction Acute kidney failure	III	Yes	No
7	8	-	Primary CD4 deficiency	Nausea/Vomiting	III	**No**	No
8	11	-	-	Nausea/Vomiting	III	**No**	No
9	43	-	-	-	-	Yes	No
10	69	Psoriatic arthritis	Golimumab	-	-	Yes	No
11	45	-	-	-	-	Yes	No
12	73	Arterial hypertension Dyslipidemia Psoriasis	Antihipertensive Hipolipemiant Adalimumab	-	-	Yes	No
13	36	Primary CD4 deficiency	-	-	-	Yes	No
14	42	-	-	-	-	Yes	No
15	6	-	-	-	-	Yes	No
16	31	-	-	-	-	Yes	No
